# Two experimental designs generate contrasting patterns of behavioral differentiation along a latitudinal gradient in *Lestes sponsa*—Common‐garden not so common after all?

**DOI:** 10.1002/ece3.6686

**Published:** 2020-08-27

**Authors:** Maria J. Golab, Tomas Brodin, Szymon Sniegula

**Affiliations:** ^1^ Institute of Nature Conservation Polish Academy of Sciences Kraków Poland; ^2^ Department of Wildlife, Fish and Environmental Studies Swedish University of Agricultural Sciences (SLU) Umeå Sweden

**Keywords:** behavioral correlations, common‐garden, latitudinal gradient, *Lestes sponsa*, native rearing conditions

## Abstract

Understanding why and how behavioral profiles differ across latitudes can help predict behavioral responses to environmental change. The first response to environmental change that an organism exhibits is commonly a behavioral response. Change in one behavior usually results in shifts in other correlated behaviors, which may adaptively or maladaptively vary across environments and/or time. However, one important aspect that is often neglected when studying behavioral expressions among populations is if/how the experimental design might affect the results. This is unfortunate since animals often plastically modify their behavior to the environment, for example, rearing conditions. We studied behavioral traits and trait correlations in larvae of a univoltine damselfly, *Lestes sponsa*, along its latitudinal distribution, spreading over 3,300 km. We compared behavioral profiles among larvae grown in two conditions: (a) native temperatures and photoperiods or (b) averaged constant temperatures and photoperiods (common‐garden). We hypothesized latitudinal differences in behavioral traits regardless of the conditions in which larvae were grown, with northern populations expressing higher activity, boldness, and foraging efficiency. When grown in native conditions, northern larvae were bolder, more active and more effective in prey capture than central and low latitude populations, respectively, as well as showed the strongest behavioral correlations. In contrast, larvae reared in common‐garden conditions showed no differences between regions in both individual traits and trait correlations. The results suggest different selective pressures acting on the studied traits across populations, with environment as a central determinant of the observed trait values. Common‐garden designed experiments may evoke population‐dependent levels of plastic response to the artificial conditions and, hence, generate results that lack ecological relevance when studying multi‐population differences in behavior.

## Graphical abstract

Damselfly larvae show plastic behavioral response to different environmental conditions along latitudinal gradient. Common‐garden experiments show fewer differences than native conditions. Our results suggest that artificial/averaged rearing conditions may generate erroneous results in behavioral study on geographically distant populations.

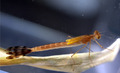

## 1 INTRODUCTION

Over the last century, animals have been faced with the challenge of coping with environments under increasingly rapid change. Adapting to such change can be problematic since environmental change often, at least in periods, occurs at a faster rate than evolutionary adaptation (Etterson & Shaw, 2001; Parmesan, 2006). Hence, animals have had to rely on other means to prevail and survive in a changing environment. As such, the first response to environmental change is usually a plastic phenotypic adjustment to the current conditions, and in most animals, behavioral plasticity is commonly the first response to environmental change (Candolin & Wong, 2012). Behavior consists of more or less genetically and functionally correlated traits that may adaptively or maladaptively vary across time and environments (Pruitt et al., 2010; Sih, Bell, & Johnson, 2004). Adaptive change in one behavior usually results in shifts in other correlated behaviors, shifts that may or may not be adaptive (Laughlin & Messier, 2015). Behavioral correlations can have strong ecological and evolutionary consequences, but has often been overlooked in favor of single traits (Sih et al., 2004). However, studies that take both single behavioral traits and behavioral correlations in multiple environments and across populations into account (Bell, 2005) might be useful for predicting future species responses to environmental change.

Abiotic factors, such as thermal conditions, changing along geographic gradients have been shown to influence life‐history traits and can also affect behavioral correlations (Roff, 1992; Sniegula, Golab, Drobniak, & Johansson, 2018). This is especially so in ectothermic organisms whose metabolism is temperature dependent (Clarke & Fraser, 2004; Wieser, 1973). For example, variation in thermal conditions influence foraging and activity rate in insects (Matthews & Matthews, 2010). Assuming that different environmental conditions may, and often do, result in different selective pressures (“adaptive hypothesis,” Bell, 2005; Dingemanse et al., 2007; Wilson, 1998), it is expected that behavioral correlations covary with life‐history traits across environmental gradients to overcome constraints linked to seasonal time limitations (Dmitriew, 2011; Foster, 1999; Sniegula et al., 2018).

Geographical gradients govern the seasonal time window available for growth, development, and reproduction of many species (Dmitriew, 2011). In the Northern Hemisphere, thermal and seasonal time constraints increase toward the north, and as a consequence, ectothermic organisms show variation in life history, morphology, and physiology across latitudes (Lindgren & Laurila, 2010; Sniegula, Janssens, & Stoks, 2017). For example, insects that are plastic in their generation‐time (voltinism) commonly take an extra season to complete a generation and show slower growth and development further north. This strategy results in larger size at emergence (Arnett & Gotelli, 2003; Johansson, 2003), which is often associated with increased reproductive success (Honěk, 1993; Sokolovska, Rowe, & Johansson, 2000) and performance in lower temperatures (Nielsen & Papaj, 2015; Stevenson, 1985, but see Golab, Johansson, & Sniegula, 2019). However, the opposite pattern can also occur if species with fixed voltinism are seasonally constrained, and compensate for this by increasing development and growth rate at northern latitudes. Such trade‐off results in smaller size at emergence (Blanckenhorn & Demont, 2004; Sniegula, Golab, & Johansson, 2016) and elevated mortality rate prior to maturation (Dańko, Dańko, Golab, Stoks, & Sniegula, 2017). Based on this, it is likely that the latitudinal compensation in life‐history traits is connected with behaviors such as foraging rate, mobility and boldness, and potential behavioral correlations between these traits. The listed behaviors have repeatedly been shown to correlate positively with resource acquisition and hence growth and development rates (Brodin & Johansson, 2004, Santostefano, Wilson, Niemelä, & Dingemanse, 2017, but see Adriaenssens & Johnsson, 2011; Debecker, Sanmartín‐Villar, de Guinea‐Luengo, Cordero‐Rivera, & Stoks, 2016).

The majority of laboratory‐based behavioral studies are performed under common‐garden conditions that, depending on the focus of the study, can sometimes mislead the interpretation of the results. On one hand, such studies might reveal a genetic background of the studied traits. On the other hand, laboratory‐bred animals and artificial conditions usually represent ecologically unrealistic situations, and as such, the results may lack ecological relevance (Archard & Braithwaite, 2010). Most temperate ectotherms use temperature and photoperiod (thermo‐photoperiod) as reliable environmental cues for estimation of seasonal time progression, and hence adjust life history and physiology accordingly (Bradshaw & Holzapfel, 2007; Gracceva et al., 2014; Sniegula et al., 2012). It is therefore important to consider these environmental variables in behavioral experiments. Hence, in order to understand the principles that rule the relationship between ecological gradients and behavioral profiles, we should aim at combining experimental designs that mimic natural environmental conditions and unravel the genetic background.

**FIGURE 1:**
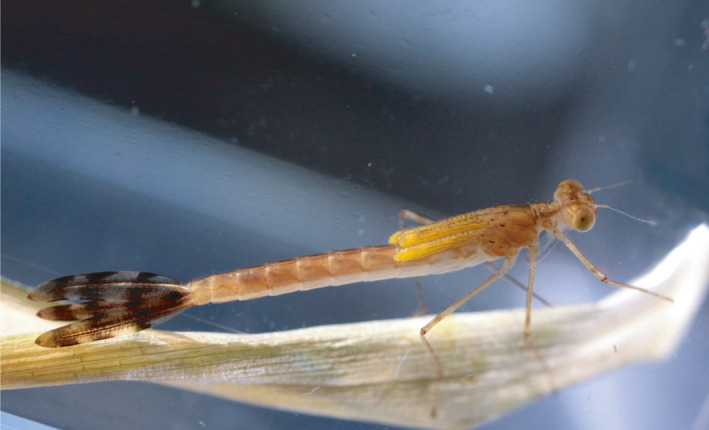
*Lestes sponsa* larvae, final instar

Here, we present results from a behavioral multi‐population study along the model species north–south distribution gradient, where the differences in individual behavioral traits: boldness, walking distance, moves, mobility (average move length), strikes (toward a prey), captures (prey captures), and correlations between these traits, were compared among: northern (Sweden), central (Poland), and southern (France) European regions (Table 1). We use two treatments: (a) native, using natural seasonal changes in thermo‐photoperiods for each of the three regions, and (b) common‐garden conditions with average, static thermo‐photoperiod. This approach aims at controlling for the possible differences arising from the two experimental designs. We use larvae of the damselfly *Lestes sponsa* (Figure 1) which is a common model species in ecological and evolutionary research (Cordoba‐Aguilar, 2009). This damselfly has a fixed one year life cycle, a wide latitudinal distribution (Dijkstra, 2006) and exhibits substantial clinal variation in life‐history traits, physiology (Sniegula et al., 2018; Sniegula, Janssens, et al., 2017) as well as larval cannibalism rates (Sniegula, Golab, & Johansson, 2017) along a latitudinal gradient. On the basis of such variation, and in line with the “adaptive hypothesis” (e.g., Bell, 2005), we suggest the presence of corresponding latitudinal variation in individual behavioral traits and behavioral correlations in *L. sponsa* larvae. Because damselfly larva invests energy primarily in development and growth (Corbet, 1999), we study traits that are particularly important for those life‐history characters. We hypothesize that (1) when grown in native thermo‐photoperiods, high latitude larvae will be bolder, more mobile, and more effective in prey capture compared to central and low latitude individuals because they experience the strongest seasonal time constraint. (1.1) Behavioral correlations in native conditions will be most pronounced toward the northern range margin as a consequence of strong directional selection for behavioral traits. (2) When grown in common‐garden conditions, that is, static thermo‐photoperiod, high latitude larvae will be bolder, more active in moving, and more effective in prey capture compared to central and low latitude individuals. (2.1) Behavioral correlations in common‐garden will be strongest in larvae from northern range margin.

**TABLE 1:** Field sampling, egg incubation data, and experimental period length of *Lestes sponsa* from northern, central, and southern regions

Data type	Northern region	Central region	Southern region
Field sampling			
Sampling date	12 Aug 2015	5 Aug 2015	15 June 2015
Sampling sites	65°36ʹN, 22°7ʹE 66°36ʹN, 19°52ʹE	53°29ʹN, 16°30ʹE 53°38ʹN, 16°22ʹE	43°29ʹN, 4°48ʹE 43°31ʹN, 4°46ʹE
Egg incubation			
Temperature [°C]	19.2	21	24.8
Photoperiod [L‐D]	20:57–3:03	17:38–6:22	16:31–7:29
Wintering start	29 Aug 2015	22 Aug 2015	2 July 2015
Wintering end	16 Oct 2015	11 Oct 2015	21 Aug 2015
Wintering time [days]	48	50	50
Egg hatching date	27 Oct 2015	16 Oct 2015	24 Aug 2015
Experimental period			
Native conditions	18 Dec 2015–9 Jan 2016	3 Jan 2016–28 Jan 2016	10 Nov 2015–19 Jan 2016
Common‐garden conditions	12 Dec 2016–7 Jan 2016	13 Dec 2015–9 Jan 2016	2 Dec 2015–17 Dec 2015

Multi‐population comparisons accounting for different experimental conditions (natural and common‐garden) will allow us to test how the choice of methodology can affect the interpretation of behavioral phenotype differences across geographical gradients. The obtained results can also be extrapolated to explain proximate causes of shifts in life‐history traits across latitudes in species with life cycles similar to *L. sponsa*. Further, our conclusions can be helpful in (a) managing the conservation of species affected by the climate change and (b) detecting environmental change at an early stage, since behavioral change is one of the first organism's reactions to environmental disturbances.

## 2 METHODS

### 2.1 Study species

The damselfly *L. sponsa* is a univoltine (one generation/season) insect with freshwater larval stage (duration of 2–3 months) and terrestrial adult stage (duration of couple of weeks; Jödicke, 1996). Females oviposit eggs during summer, and the egg is the overwintering stage. The European latitudinal distribution of *L. sponsa* ranges from northern Sweden to northern Spain and southern France (Dijkstra, 2006; Sniegula et al., 2016).

### 2.2 Field sampling

To collect a representative sample from each population and lower the impact of site‐specific environmental effects, we collected 20 egg clutches from two replicate populations (sampling sites in Table 1) per region using a well‐established method (Sniegula, Drobniak, Golab, Johansson, 2014, Figure 2a, Table 1). Low and high latitude sampling sites are at the species southern and northern range margins, respectively (Dijkstra, 2006). Detailed description of population densities is described in Sniegula, Golab, Drobniak, and Johansson (2016), but in general, high latitude populations contain fewer individuals than central and low latitude populations.

**FIGURE 2:**
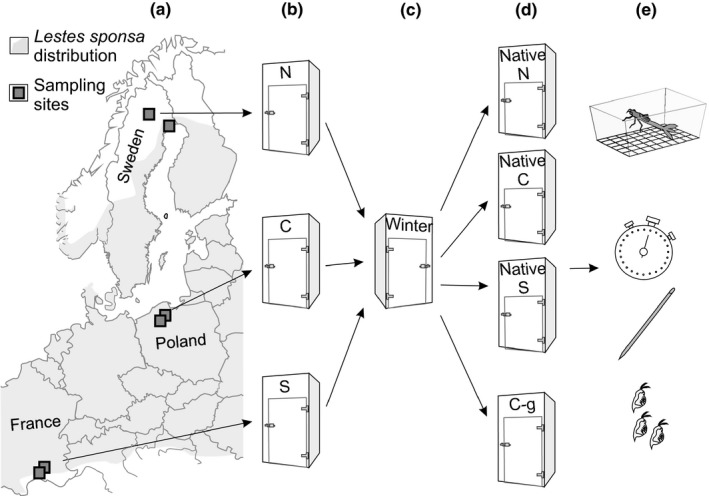
Sampling sites (a), egg incubation (b), wintering (c), laboratory rearing in native and common‐garden (C‐g) conditions (d) and behavioral experiments (e) of *Lestes sponsa* larvae. N—northern, C—central, S—southern breeding conditions. Map of the European distribution of *L. sponsa* modified from Dijkstra (2006)

### 2.3 Experimental setup

After arrival to the laboratory, the eggs were placed in plastic containers filled with 250 ml of dechlorinated tap water and placed in climate chambers with thermo‐photoperiods simulating summer conditions at each latitude of origin of the populations, that is, the environmental conditions at the collection sites (Figure 2b, Table 1). Seventeen days after the eggs were placed in the climate chambers, simulated winter conditions were initiated. Temperature was lowered to 15°C while maintaining the existing photoperiod regime. On the following day, the temperature was reduced to 5°C and the photoperiod set to 24 hr of darkness in order to simulate winter conditions (Figure 2b, Table 1). Immediately after the end of the simulation of winter conditions that took slightly different number of days for the latitudes sampled (Table 1), all eggs were transferred to another climate chamber with temperature 21.9°C and photoperiod L‐D 19:25–4:35. This photoperiod reflected the longest day length at the central latitude across our sampling latitudinal gradient, 55°N, and would trigger synchronous hatching. Also, individuals from each latitude experience this temperature for at least several hours a day during the growth season in nature (Sniegula, Golab, Drobniak, et al., 2016, Figure 2c). Immediately after hatching clutches originating from each population were mixed and then larvae were randomly placed individually in round plastic containers (diameter 7 cm, height 4 cm) filled with dechlorinated tap water. Hence, from this step, individuals were followed at the population level, not clutch/individual level. Larvae were fed twice a day with *Artemia* nauplii (mean nauplii portion: 121.83, *SD* = 9.48, *N* = 10) until behavioral observations started, that is, three days after the larvae had moulted into the final instar. Individual larvae were reared under one of two conditions: native latitude conditions or common‐garden conditions (Figure 2d).

### 2.4 Native conditions

**FIGURE 3:**
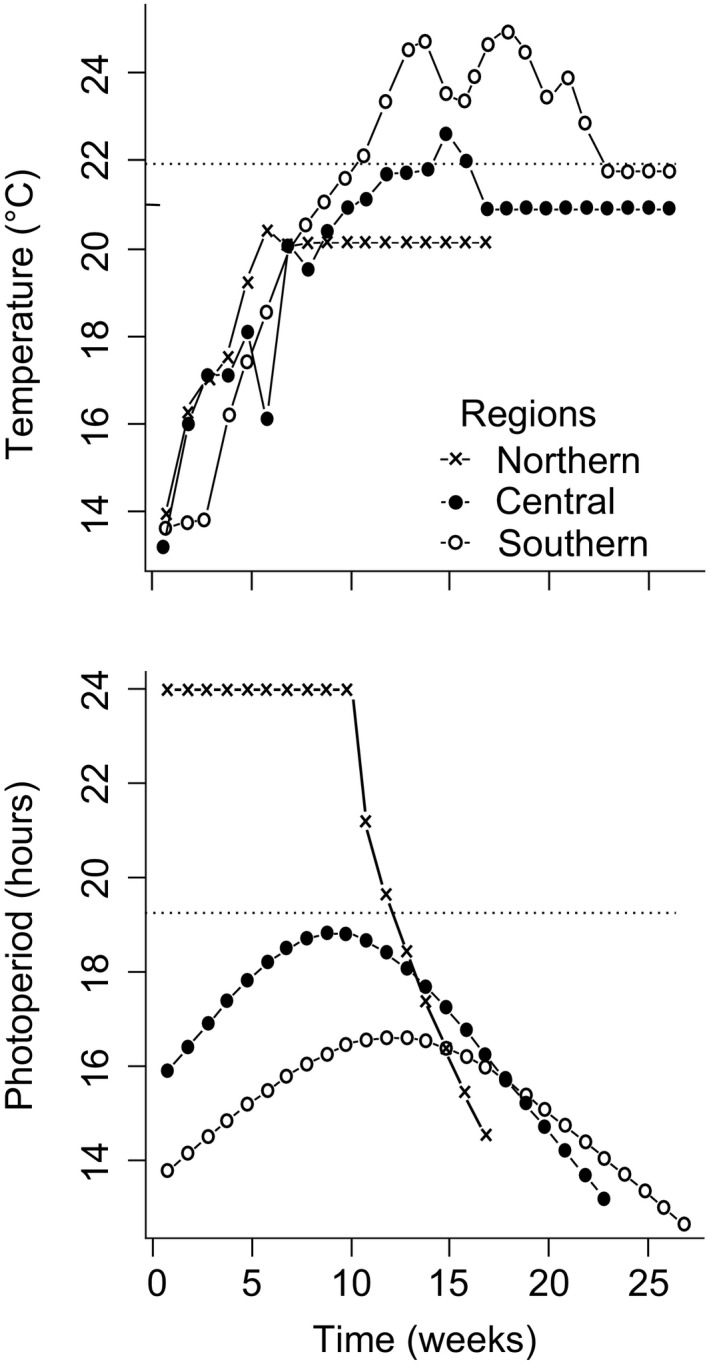
Temperature and photoperiod used during the laboratory experiment on larvae of *Lestes sponsa* originating from three different latitudinal regions: Northern (Sweden), central (Poland), and southern (France)

On the day of hatching, 80 hatchlings from both populations within each latitude were randomly chosen. This generated a total of 480 larvae at the start of the native condition experiment. The larvae were moved to, and reared in, three climate chambers programmed for conditions matching either high, central, or low latitude temperature and photoperiod (Figures 2d and 3). On the day of the transfer, thermo‐photoperiods for high, central, and low latitude conditions matched May 30 (temp. 14°C), April 25 (temp. 13.3°C), and April 4 (temp. 13.8°C) for low latitude, respectively. We chose these dates because then the temperatures derived from FLake model were >10 C degrees in every studied latitude. It was earlier indicated by Corbet (1956) that temperature > 10 C degrees triggers *Lestes sponsa* egg hatching (postdiapause spring hatching). To follow natural changes in thermo‐photoperiod in the different latitudes, we adjusted temperature and light accordingly once a week (Figure 3). Photoperiod was extended by adding morning and evening Civil twilight, and this is because insects are very sensitive to light intensity and the threshold at which they register light is very low (Saunders, 2002).

Three days after larvae entered last instar (when exoskeleton hardened, Figure 1), behavioral observations started. On the day of the walking observation, each larva was moved from the climate chambers and individually placed in bigger containers (12 × 8 × 5 cm high) filled with dechlorinated tap water, with a grid of 2 × 2 cm drawn on the bottom (Figure 2e). These containers were placed in an air‐conditioned room, where the air temperature was comparable (±0.1°C) to the temperature set in the climate chambers at the time of behavioral observations. Also, the water used for the behavioral observations was previously stored in the given climatic chamber in order to observe the larvae under similar water conditions as in the chamber. Before the observations started, larvae were allowed to acclimate to new containers for 30 min. Behavioral trials were initiated by larva being poked in the tail (caudal lamella) with a thin wooden stick that triggered larval escape response followed by a freeze period (inactivity). Latency of larva to start moving following freezing was used as a proxy of boldness; the shorter the freeze period the bolder the larvae. Following this, larva was left undisturbed for 3 min in order to calm down, after which 10 laboratory cultured *Daphnia* sp. of standardized size were introduced to the container. Then, the feeding behavior of larvae was monitored for 15 min. During the feeding trial, the observer noted: number of times the larva moved (moves), total distance the larva traversed (walking distance), number of strikes, number of prey captured (capture), and number of prey eaten (prey). Movements were scored when separated by at least 2 s of larval immobility.

### 2.5 Common‐garden conditions

After hatching, 24 larvae per population were randomly chosen to be reared in the same chamber as where they had hatched, in constant temperature of 21.9°C and constant photoperiod L:D 19:25–4:35. This generated 48 larvae per region and a total of 144 larvae at the start of the common‐garden experiment. Both rearing conditions and behavioral trials were identical to those described in native conditions experiment (Figure 2d,e).

### 2.6 Statistical analyses

All statistical analyses were performed in IBM Statistics SPSS v.24. To check for differences between regions in individual traits (walking distance, moves, strikes, captures and prey eaten and boldness), we run separate GLMM models for native and common‐garden conditions. Region, sex, and interaction between the two were independent variables; population within region was used as a random effect. Nonsignificant interactions between region and sex were removed from the final models. We ran two separate principal component analyses (PCA) with varimax rotation in two treatments: native and common‐garden conditions, in order to assess the individual behavioral trait structure and find behavioral correlations. The cutoff for extracted components deemed to contribute meaningfully was set to eigenvalue > 1. To check for between regions differences in correlations between activity and foraging syndrome (here, we used PCA scores), we used separate GLMM models. Region, sex, and interaction between the two were independent variables. Population within region was a random effect. Nonsignificant interactions between region and sex were removed from the final model.

Part of the data set is also presented in an article where we focus explicitly on cannibalism rate in *L. sponsa* (Sniegula et al., 2016).

## 3 RESULTS

**FIGURE 4:**
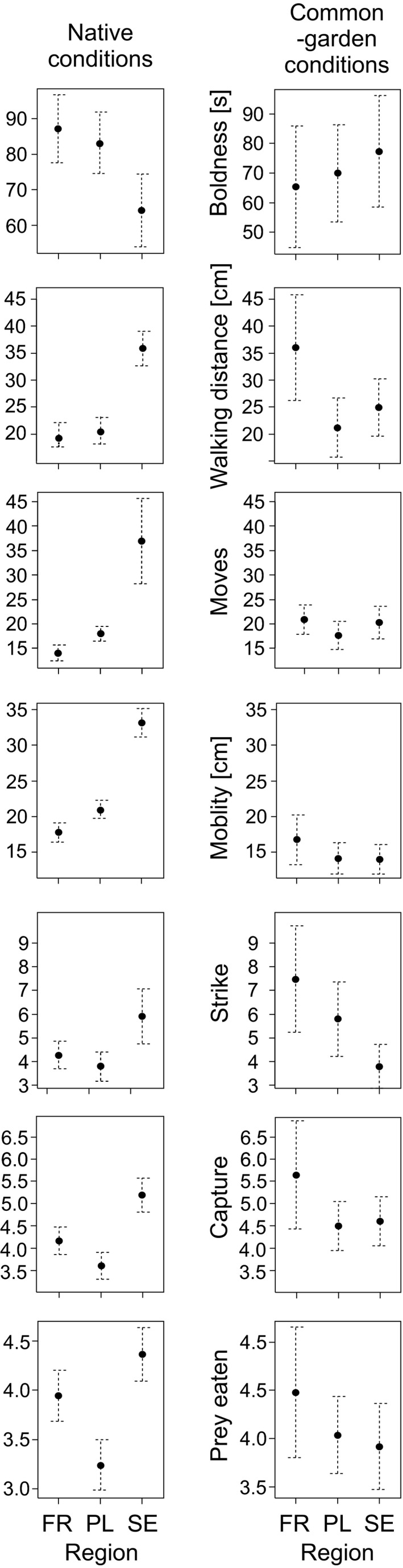
Mean (CI 95%) behavioral traits in *Lestes sponsa* larvae from northern (SE), central (PL), and southern (FR) regions. Boldness declines with increasing time because low time scores (short freezing time) indicate high boldness

Under native conditions, Swedish (northern) larvae always had the highest values of boldness, walking distance, moves, strikes, captures, and prey eaten, followed by French (southern) and Polish (central) larvae. Polish region larvae had intermediate and French the lowest values of moves; however, larvae from the two regions did not differ in boldness, walking distance, strikes, captures, or prey eaten (Figure 4, Table 2). In addition, there was a behavioral difference between the sexes: Females were bolder and had higher number of strikes (close to significant, *p* = .052) than males.

**TABLE 2:** GLMM for difference of individual behavioral traits between regions in native and common‐garden rearing conditions

Trait	Predictor	*χ* ^2^	*df*	*p*
Native conditions				
Boldness	Region	10.491	2	**.005**
	Sex	4.099	1	**.043**
Walking distance	Region	89.516	2	**<.001**
	Sex	0.146	1	.703
Moves	Region	44.051	2	**<.001**
	Sex	0.928	1	.335
Mobility	Region	213.98	2	**<.001**
	Sex	0.055	1	.813
	Region*Sex	8.684	3	**.013**
Strikes	Region	14.757	2	**<.001**
	Sex	3.787	1	**.052**
Captures	Region	47.100	2	**<.001**
	Sex	0.319	1	.572
Prey eaten	Region	38.355	2	**<.001**
	Sex	1.007	1	.316
Common‐garden conditions				
Boldness	Region	0.856	2	.651
	Sex	4.624	1	.099
Walking distance	Region	8.729	2	**.013**
	Sex	1.641	1	.440
Moves	Region	0.915	2	.633
	Sex	2.569	1	.276
Mobility	Region	1.029	2	.597
	Sex	2.558	1	.278
Strikes	Region	9.948	2	**.007**
	Sex	4.356	1	.113
Captures	Region	5.233	2	.073
	Sex	1.512	1	.469
Prey eaten	Region	2.738	2	.254
	Sex	2.239	1	.326

Note

*p*‐Values < .05 are in bold. Only significant interactions are shown.

In contrast, under common‐garden conditions, there were no significant differences between the three regions in moves, captures, and prey eaten. However, Swedish larvae walked longer than Polish larvae, and French larvae carried out significantly fewer strikes compared to Swedish larvae (Figure 4, Table 2).

The PCA analysis divided the traits into two components in native conditions: One behavioral correlation connected to activity and boldness (walking distance, moves, boldness; hereafter called activity syndrome) and the second connected to foraging traits (strikes, prey capture, prey eaten; hereafter called foraging syndrome), which cumulatively explained 62.14% of the total variance of the original variables (Table 3). In common‐garden conditions, PCA extracted three components: activity syndrome, foraging syndrome, and third component were related to boldness only, explaining 83.26% of total variation (Table 3).

**TABLE 3:** Principal component analysis of behavioral scores from native and common‐garden conditions

Behavioral trait	Native conditions		Common‐garden conditions		
	PC1	PC2	PC1	PC2	PC3	
Eigenvalue	3.017	1.332	2.863	1.924	1.041	
Proportion of variance	43.106	19.031	40.906	27.484	14.869	
Boldness	−0.377				0.974	
Walking distance	0.907		0.916			
Moves	0.656		0.981			
Mobility	0.900		0.982			
Strikes		0.470		0.643		
Captures		0.906		−0.869		
Prey eaten		0.942		0.863		

**FIGURE 5:**
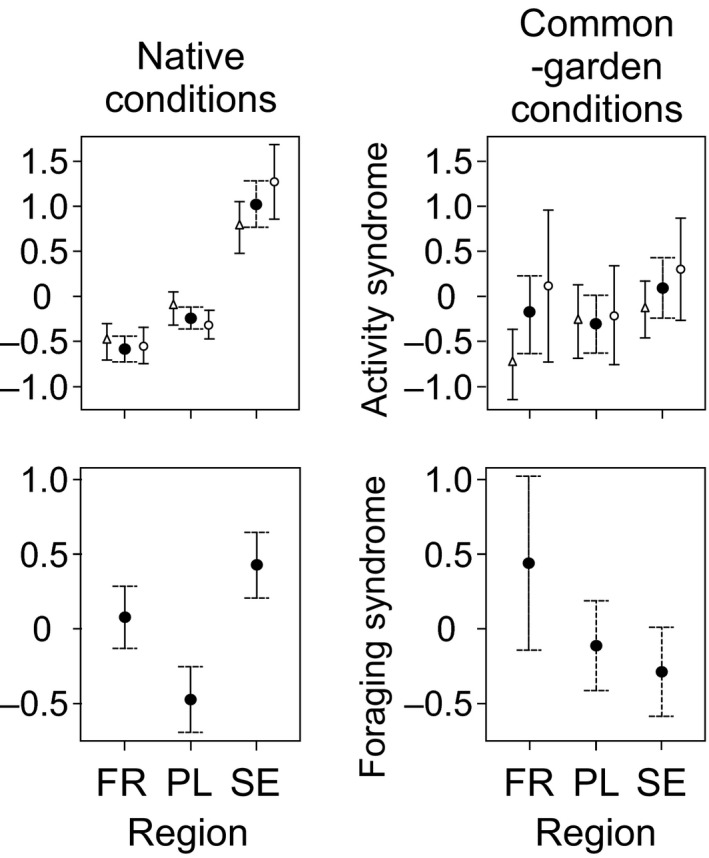
Mean (CI 95%) behavioral trait correlations (i.e., component scores; black circles) of *Lestes sponsa* larvae from northern (SE), central (PL), and southern (FR) regions and mean values for females (white circles) and males (triangles)

In native rearing conditions, Swedish larvae had the highest values of both behavioral correlations (Figure 5, Table 4). Polish larvae had intermediate values of activity syndrome and lowest values of foraging syndrome, whereas French larvae had intermediate values of foraging syndrome and lowest activity syndrome. The interaction between region and sex was significant for the activity syndrome with French females having lower syndrome than males, and Swedish females having higher values of activity syndrome (Figure 5). In common‐garden conditions, neither of the behavioral correlations differed between regions, but there was a significant difference between sexes in the activity syndrome with higher activity shown by females (Figure 5, Table 4).

**TABLE 4:** GLMM for activity and foraging syndromes between three regions in native and common‐garden breeding conditions

Behavioral correlation	Predictor	F	*df*	*p*
Native conditions				
Activity syndrome	Region	80.512	2	**<.001**
	Sex	0.769	1	.382
	Region*Sex	4.203	2	**.016**
Foraging syndrome	Region	12.581	2	**<.001**
	Sex	0.507	1	.477
Common‐garden conditions				
Activity syndrome	Region	1.307	2	.278
	Sex	4.739	1	**.033**
Foraging syndrome	Region	2.191	2	.120
	Sex	3.072	1	.085

Note

*p*‐Values < .05 in bold.

## 4 DISCUSSION

Here, we present results from a multi‐population large‐scale study of how key environmental factors along a latitudinal gradient affect ecologically important behaviors and how this in turn depends on experimental setup. In general, we found latitudinal behavioral differences that supported our hypothesis (1) and (1.1), but rejected hypotheses (2) and (2.1). When larvae were grown in native thermo‐photoperiod, the more time constrained high latitude populations differed from less time constrained central and low latitude populations in all individual traits measured and behavioral correlations. When individuals were grown in common‐garden conditions, with all populations experiencing the same constant thermo‐photoperiod, the expression of individual behavioral traits and behavioral correlations in most cases did not differ between regions. Altogether, our results suggest that behaviors related to activity and foraging are plastic, because larvae reared under common‐garden conditions showed fewer differences among geographically distant populations than larvae reared under native conditions. This in turn points at the importance of choosing the appropriate experimental design when studying behavior across latitudinal gradients. Experiments based on artificial/averaged conditions may generate erroneous results and as a consequence flawed conclusions.

Geographically predictable variation of behavioral traits across different populations in nature is commonly attributed to the combined effects of latitudinally varying environments and genotypes (Foster & Endler, 1999). In our study, high latitude populations were bolder and moved more than central and low latitude populations, respectively, when reared in native conditions, which confirmed our hypothesis (1). This differentiation is presumably a response to a shorter growth season and lower ambient temperatures in the north (Cahill et al., 2014; Clarke & Gaston, 2006). In addition, central damselflies both captured and ate significantly less prey when compared to southern ones. This pattern can be explained in means of species range margins (Gaston, 2009). Peripheral populations are subjected to least favorable environmental conditions, leading to behavioral adaptations (e.g., increased boldness, activity, Gaston, 2009). Contrastingly, central populations usually live under less stressful conditions, especially in terms of time window available for growth and development (Sagarin, Gaines, & Gaylord, 2006; Williams, Ives, & Applegate, 2003), allowing them to express less extreme values of behavioral traits and still mature prior to the end of the breeding season.

Interestingly, the trend of central damselflies expressing the lowest, and southern populations showing intermediate trait values has also been found in mating behavior of adult *L. sponsa* studied in seminatural conditions (Golab et al., 2019).

An alternative, but not exclusive, explanation for intermediate expressions of behavior in southern populations reared in native conditions could be that under some circumstances damselflies, from these populations, may complete a second generation within a season (Philip Lambret, personal communication). Higher activity, foraging rate, and boldness lead to increased prey capture and as a consequence higher energy gain (Brodin, 2009; Hassell & Southwood, 1978). Higher energy acquisition and investment into development could allow southern larvae to speed up development to complete an additional generation within a season, given summer‐dry Mediterranean climate is not imposing additional environmental constraint—drought. Note that bivoltinism in southern populations should be frequent enough to be evolutionary relevant (e.g., Lopez‐Villalta, 2010). Also, heat stress can impact damselfly behavior in southern populations. It has been proposed that high air temperature (exceeding 31 degrees) reduces *L. sponsa* mating activity (Golab et al., 2019). If this is the case, it would explain why individuals from the southern populations are more time constrained than individuals from the center, yet less so than those from the north (cf. DeBlock, Pauwels, Van Den Broeck, De Meester, & Stoks, 2013). Another evidence supporting the “adult heat stress” hypothesis comes from Japanese populations. In these populations, bivoltinism is excluded by an adult prereproductive diapause (Uéda, 1989).

In addition to measuring individual behavioral traits, we also focused on trait correlation, that is, traits that covary across time and space (Fischer, Ghalambor, & Hoke, 2016; Lande & Arnold, 1983). Our analyses of the behavioral measurements in native conditions revealed two significant axes of behavior. The first axis represented a foraging syndrome and the second axis an activity syndrome. In line with the prediction of hypothesis (1.1), we found geographic differentiation in both foraging and activity syndrome and boldness in larvae reared under native conditions. Swedish larvae had always more pronounced behavioral correlations than larvae from French populations. Polish populations scored significantly lower on the foraging syndrome but higher in activity syndrome compared to the French populations. As far as we know, this is the first study presenting data on latitudinal effects on behavioral correlations in an invertebrate. Earlier study on a vertebrate has shown for instance that a boldness–activity and boldness–exploration syndrome in skink (*Lampropholis delicate*) varied geographically. However, the authors did not suggest what environmental factors caused the differences (Michelangeli, Chapple, Goulet, Bertram, & Wong, 2019). The reason for foraging and activity syndromes being most pronounced at high latitude populations are most likely because of the strong seasonal time constraints these populations are exposed to (Brown, Stevens, & Kaufman, 1996; Cahill et al., 2014; Sniegula et al., 2018). The mechanisms for this have already been discussed above with regard to individual behavioral traits. Another explanation could be that traits profiling activity or prey capturing often are tightly linked to the energetic state, and as such are environmentally sensitive, and therefore more likely to be less stable than traits constrained by physiology or morphology (Castellano, Cuatto, Rinella, Rosso, & Giacoma, 2002; Smith & Hunter, 2005).

Predation is an important biotic factor which influences local adaptive processes and consequently may shape behavioral profiles (Bell, 2005; Bengston & Dornhaus, 2014; Herczeg, Gonda, & Merilä, 2009). Both increased boldness and activity has been previously shown to increase predation risk, including cannibalism, (Brodin & Johansson, 2004; Sniegula, Golab, et al., 2017); hence, our range‐margin populations (northern and potentially also southern) are probably forced to accept higher risks of predation (growth‐predation risk trade‐off, McPeek, 2004; Werner & Anholt, 1993) in the wild, compared to central ones. However, our experiments were controlled for predation risk (including cannibalism), and hence, this factor is of negligible importance in our results. But still, we do not have data on predation risk at source populations where the eggs were collected. Therefore, future studies should consider collecting data on predation risk in the source populations and incorporate it into the experimental design in order to test the influence of this important biotic factor on both single behavioral expressions and behavioral syndromes (Briffa, 2013).

To conclude, our study conducted under native conditions is in line with previous findings suggesting that local environmental pressures shape behavioral profiles in animals (Foster & Endler, 1999), and it highlights the fact that species range margins can be of special interest for studying the connection between environmental factors and behavioral traits and trait correlations. This is very important because peripheral populations often show strong adaptation to local environments (for review see: Kawecki, 2008), and thus may be most vulnerable to environmental change.

Our results suggest that hypothesis (2), assuming differences in behavioral traits across latitudes in common‐garden conditions, should be rejected. One commonly used interpretation of such results is that the genetic component, that shapes trait expressions, alone is less important than its interaction with environmental effects (phenotypic plasticity and epigenetics; Pigliucci, 2001). Indeed, phenotypic plasticity in behaviors may be the central determinant of the observed trait values (Dingemanse, Kazem, Réale, & Wright, 2010; Forsman, 2015; Tuomainen & Candolin, 2011).

The common‐garden experiment elicited a plastic response of the larvae to the artificial conditions. This is not surprising since *L. sponsa* larvae regulate its life cycle by means of environmental signals such as photoperiod (Norling, 2018; Sniegula & Johansson, 2010). Within a geographic gradient, increasingly longer days cue individuals to develop, grow, and emerge faster in order to complete the life cycle prior to the end of the season (Sniegula et al., 2018; Sniegula & Johansson, 2010). At high latitudes summer, photoperiods are very long and may even consist of continues light (24 hr). In our common‐garden conditions, the constant day length (L‐D 19:25–4:35) probably indicated a late‐season photoperiod at hatching, yet not to the same degree for individuals from all populations and larval sizes (stadia). It simulated high, intermediate, and low seasonal time constraint for southern, central, and northern populations, respectively. Photoperiod cues larval development when individuals are in intermediate and late stadia; early stadia are least sensitive in response to different photoperiods (Norling, 2018). Therefore, if behavioral responses in common‐garden were driven predominantly by phenotypic plasticity, southern individuals would have expressed highest values of the behavioral traits connected to energy acquisition (e.g., boldness, moves, and prey eaten). However, this was not the case, probably because damselflies were subjected to these constant conditions for unnaturally long time (Figure 2) and/or because time constraints are not the only environmental factor that shapes behavioral traits. Future across latitude full‐factorial experiments will help to explain this pattern even further. Altogether, constant photoperiod, commonly used in common‐garden experiments, can make it difficult to find and/or understand differences among latitudinally distant populations.

We also found no differences in the expression of behavioral correlations (three axes: activity syndrome, foraging syndrome, and boldness alone, Table 3) among the three studied regions of damselflies reared in common‐garden conditions, rejecting hypothesis 2.1. As already stated, *L. sponsa* is a species that is highly dependent on abiotic factors like photoperiod as environmental cues. Therefore, results from multiple‐population studies in common‐garden experiments are likely biased by the population‐dependent difference between native and experimental conditions. This reasoning is supported by the fact that common‐garden conditions can act as unfamiliar environment for our model species, which may induce plastic behavioral responses.

We found a significant difference between sexes in activity syndrome in common‐garden rearing conditions where females were more active than males. The lower activity of males could potentially be explained by sexual size dimorphism. Females are larger than males at emergence (Sniegula et al., 2016) and hence have to gain more energy to grow larger, and as such are forced to be more active than males (Johansson, Crowley, & Brodin, 2005).

Day length is an important cue to regulate and synchronize reproduction and associated events in vertebrates (Bunyaga & Makungu, 2018; Jain & Kumar, 1995). In the era of climate change, many species expand their range to track suitable habitat and/or are forced to live in and adapt to novel environmental conditions (Hickling, Roy, Hill, Fox, & Thomas, 2006; Parmesan, 2006). Species expanding northward experience for example longer days during summer, but also lower temperatures. Testing behavioral reactions to such new conditions may allow for predicting shifts in, for instance, foraging activity or boldness which may affect important life‐history characters such as growth rate, clutch size, and/or mortality rates (e.g., Cardona et al., 2014; Stamps, 2007; Vollrath, 1987). For such purposes, the experimental setup must be carefully considered when planning behavioral studies on animals originating from geographically distant populations.

To summarize, our study confirms plastic behavioral response to environmental conditions and, for the first time, shows a latitudinal effects on behavioral correlations in an invertebrate, with the highest trait values for the northern, most time constrained, populations. Presented results also confirm that population level behavioral correlations may predict individual level behaviors (Pruitt et al., 2010). Further, we suggest that a caution should be taken when interpreting behavioral measures obtained in common‐garden conditions. This is because the difference between native and common‐garden conditions often varies between geographically distant populations. Individuals from different populations reared in artificial/averaged conditions are hence subjected to different treatments relative to their native conditions, despite the common‐garden set‐up. Finally, study‐site location, in relation to the species range margins, should be considered when studying behavioral profiles. Altogether, our results provide important guidance for the conservation of invertebrate species affected by environmental change as well as for designing and interpreting behavioral experiments on geographically distant populations.

## CONFLICTS OF INTEREST

The authors declare that they have no conflicts of interest.

## AUTHOR CONTRIBUTION

**Maria J. Golab:** Conceptualization (equal); Data curation (lead); Formal analysis (equal); Funding acquisition (equal); Investigation (lead); Methodology (lead); Project administration (equal); Resources (equal); Visualization (lead); Writing‐original draft (equal). **Tomas Brodin:** Conceptualization (supporting); Data curation (supporting); Formal analysis (equal); Methodology (supporting); Project administration (equal); Software (lead); Supervision (equal); Writing‐original draft (equal). **Szymon Sniegula:** Conceptualization (equal); Data curation (equal); Funding acquisition (equal); Investigation (equal); Methodology (equal); Project administration (supporting); Supervision (equal); Validation (equal); Writing‐original draft (equal).

## Data Availability

Behavioral traits measurements, sex, treatment, and region of origin data: Dryad https://doi.org/10.5061/dryad.jdfn2z36x.
